# Functional changes in sleep-related arousal after ketamine administration in individuals with treatment-resistant depression

**DOI:** 10.1038/s41398-024-02956-2

**Published:** 2024-06-04

**Authors:** Elizabeth D. Ballard, Deanna Greenstein, Philip T. Reiss, Ciprian M. Crainiceanu, Erjia Cui, Wallace C. Duncan, Nadia S. Hejazi, Carlos A. Zarate

**Affiliations:** 1 Experimental Therapeutics and Pathophysiology Branch, National Institute of Mental Health, National Institutes of Health, Bethesda, MD USA; 2 Department of Statistics, University of Haifa, Haifa, Israel; 3 Department of Biostatistics, Johns Hopkins School of Public Health, Baltimore, MD USA; 4 Division of Biostatistics and Health Data Science, University of Minnesota Twin Cities, Minneapolis, MN USA

**Keywords:** Neuroscience, Biomarkers

## Abstract

The glutamatergic modulator ketamine is associated with changes in sleep, depression, and suicidal ideation (SI). This study sought to evaluate differences in arousal-related sleep metrics between 36 individuals with treatment-resistant major depression (TRD) and 25 healthy volunteers (HVs). It also sought to determine whether ketamine normalizes arousal in individuals with TRD and whether ketamine’s effects on arousal mediate its antidepressant and anti-SI effects. This was a secondary analysis of a biomarker-focused, randomized, double-blind, crossover trial of ketamine (0.5 mg/kg) compared to saline placebo. Polysomnography (PSG) studies were conducted one day before and one day after ketamine/placebo infusions. Sleep arousal was measured using spectral power functions over time including alpha (quiet wakefulness), beta (alert wakefulness), and delta (deep sleep) power, as well as macroarchitecture variables, including wakefulness after sleep onset (WASO), total sleep time (TST), rapid eye movement (REM) latency, and Post-Sleep Onset Sleep Efficiency (PSOSE). At baseline, diagnostic differences in sleep macroarchitecture included lower TST (*p* = 0.006) and shorter REM latency (*p* = 0.04) in the TRD versus HV group. Ketamine’s temporal dynamic effects (relative to placebo) in TRD included increased delta power earlier in the night and increased alpha and delta power later in the night. However, there were no significant diagnostic differences in temporal patterns of alpha, beta, or delta power, no ketamine effects on sleep macroarchitecture arousal metrics, and no mediation effects of sleep variables on ketamine’s antidepressant or anti-SI effects. These results highlight the role of sleep-related variables as part of the systemic neurobiological changes initiated after ketamine administration. Clinical Trials Identifier: NCT00088699.

## Introduction

The glutamatergic modulator ketamine is associated with rapid reductions in both depressive symptoms and suicidal thoughts [1, 2]. Ketamine is also associated with transient, systemic changes across a range of biomarkers at the neuroimaging, metabolic, and neurotrophic levels (for a comprehensive review, see [3]). Such changes suggest that ketamine may rapidly normalize aberrant pathophysiology associated with major depressive disorder (MDD), leading to homeostatic theories of ketamine’s mechanism of action [4]. For example, magnetoencephalography (MEG) studies found changes in gamma power—a proxy marker of balance between glutamatergic excitation and gamma aminobutyric acid (GABA)-ergic inhibition—after ketamine administration in both human studies and preclinical models [5]. Furthermore, baseline gamma power appears to moderate the relationship between gamma power changes and antidepressant response post-ketamine [6]. Dynamic causal models of neural excitation/inhibition balance—specifically increased excitatory and inhibitory coupling—have also been linked to enhanced antidepressant response [7]. In addition to these electrophysiological markers during awake states, ketamine is associated with sleep electrophysiological changes, including increased slow wave activity in the first non-rapid eye movement (NREM) sleep episode as a potential marker of synaptic plasticity and homeostatic sleep regulation [8]. Such findings have led to homeostatic theories of ketamine’s mechanism of action and highlighted the importance of sleep in maintaining antidepressant effects [9].

Sleep disturbances are common in MDD, particularly in treatment-resistant depression (TRD) [10], such that one of the diagnostic criteria for a major depressive episode is sleep difficulties. Meta-analyses of polysomnography (PSG) studies found that MDD is associated with less continuous sleep (more awakenings, longer sleep latency), less “deep” sleep (longer duration of Stage 1 sleep, shorter duration Stage 2 and slow wave sleep), and increased rapid eye movement (REM) pressure (shorter REM latency, increased REM density, and longer REM duration) [11]. Individuals with MDD are also likely to have co-morbid insomnia, which is thought to be caused by sleep-related hyperarousal or an imbalance between sleep- and wake-promoting neural circuits (ventrolateral preoptic nucleus (VLPO) and ascending reticular activating system (ARAS), respectively) that leads to nocturnal wakefulness and insomnia symptoms [12, 13]. In support of the association between sleep hyperarousal and MDD, insomnia, and depression have each been linked to increased nocturnal alpha power, a measure of quiet wakefulness, and beta power, a measure of alert wakefulness [14–17].

A growing body of research has also implicated sleep hyperarousal as a critical risk factor for suicide [18, 19]. Previous work by our group demonstrated that nocturnal wakefulness was associated with next-day suicidal thoughts [20] and that responders to ketamine with suicidal ideation (SI) demonstrated decreased nocturnal wakefulness the night after ketamine administration compared to ketamine responders without SI [21]. More recently, our group demonstrated that oscillations in overnight beta power were associated with next day SI, supporting a relationship between the sleep-related arousal process and suicide risk [22]. Despite the evidence supporting ketamine’s therapeutic effects on sleep physiology, depression, and SI, the impact of ketamine on sleep-arousal markers remains undefined; this includes temporally-dependent oscillations in arousal-related frequency bands such as alpha and beta as well as macroarchitecture measures of arousal such as sleep efficiency or time spent awake after sleep onset. Additionally, it is not known whether sleep-arousal markers represent a mediating factor with regard to ketamine’s impact on depression and SI, as many potential mechanisms have been proposed; these include dissociative side effects and earlier antidepressant response in addition to the aforementioned gamma power and slow wave sleep changes [5, 8, 23, 24].

This study sought to combine both functional data analysis as well as traditional sleep arousal measures to evaluate the impact of ketamine on measures of sleep arousal. Functional analyses were emphasized because they retain information on the temporal electrophysiological patterns integral to the sleep process. In comparison, omnibus sleep metrics rely on a sleep staging process that renders PSG data into tractable bite-size pieces but collapses the highly complex, temporally-dependent sleep process into single values [25]. Using a combined approach allows the capture of big-picture macroarchitecture without forsaking the richness of spectral data proximal to the dynamic neural processes that occur during sleep [25, 26]. To our knowledge, this a novel approach to understanding sleep in depression and the effects of rapid antidepressant interventions, such as ketamine, on sleep. In addition, by using data from a methodologically rigorous, crossover, placebo-controlled trial, the study was able to implement this approach to treatment effects on sleep at a within-person level.

This study had three primary aims. The first aim was to evaluate baseline diagnostic differences in sleep arousal measures between unmedicated individuals with TRD and healthy volunteers (HVs) who enrolled in a randomized, placebo-controlled, crossover ketamine trial [6]. After determining baseline diagnostic differences, the second aim was to evaluate the impact of ketamine on measures of sleep arousal in the TRD participants; this allowed us to determine whether ketamine normalizes sleep-related arousal in depression. The final aim was to evaluate whether ketamine’s effects on sleep-related arousal mediated reductions in SI and depressive symptomatology on the day following ketamine administration. The hypotheses were that the TRD group would have greater sleep arousal at baseline than the HV group; that ketamine would normalize and attenuate arousal in the TRD group; and that this improvement would mediate ketamine’s therapeutic effects on SI and depressive symptomatology. These hypotheses are in line with homeostatic theories suggesting that ketamine may stabilize sleep-related indicators of arousal and subsequently improve mood and SI [27].

## Materials and methods

### Participants

Participants were drawn from a previously published randomized clinical trial (NCT00088699) of intravenous ketamine (0.5 mg/kg) compared to saline placebo [6]. All patient participants were 18–65 years old and diagnosed with recurrent MDD without psychotic features using the Structured Clinical Interview for Axis I DSM-IV Disorders (SCID)-Patient Version. Treatment resistance was characterized as not responding to at least one psychiatric medication during the current depressive episode. Participants were free from psychiatric medications for at least two weeks (five weeks for fluoxetine, three weeks for aripiprazole) prior to baseline assessment and were required to have a score of 20 or more on the Montgomery-Asberg Depression Rating Scale (MADRS) [28] before each ketamine or placebo infusion. The participant sample also included HVs, who did not meet criteria for any Axis I disorders on the SCID and had no first-degree relatives with Axis I Disorders. Participants were voluntary inpatients over the course of the clinical trial. All participants were considered to be in good physical health based on medical history and physical exam.

For the current study, a subset of HVs and participants with TRD were selected who had at least one PSG recording for five hours following sleep onset (defined as the first 30-s epoch of stage 2 NREM sleep) (TRD: n = 36; 61% F; mean age = 36.4 yrs (SD = 10.29); HVs: n = 25; 64% F; mean age = 34.0 yrs (SD = 10.6)). See Table 1 for the demographic and clinical characteristics of the sample. The study was approved by the National Institutes of Health (NIH) Combined Neuroscience Institutional Review Board (NCT00088699), and all methods were performed in accordance with relevant guidelines and regulations. All participants provided written informed consent.

**Table 1:** Demographics and descriptive sleep variables averaged (SD) over both baselines for HVs (n = 25) and individuals with TRD (n = 36).

	HV	TRD
Sex		
Female	16 (64%)	22 (61%)
Male	9 (36%)	14 (39%)
Age	34.04 (10.60)	36.44 (10.29)
Clinical Characteristics		
MADRS Total	1.48(1.64)	33.33 (4.54)
HAM-D Total	1.56 (1.36)	21.78 (4.43)
SSI5 Total	0.00(0)	1.71 (1.85)
HAM-A Total	1.44(1.33)	22.72 (6.40)
Severe Early Insomnia on HAM-D item	1 (4%)	17 (47%)
Severe Middle Insomnia on HAM-D item	1 (4%)	8 (22%)
Severe Late Insomnia on HAM-D item	0 (0%)	5 (14%)
PSG Variables		
NREM Stage 1 post-sleep onset (units=minutes)	25.68 (17.98)	24.49 (11.95)
NREM Stage 2 post-sleep onset (units=minutes)	259.61 (35.40)	229.29 (39.07)
NREM Stage 3 or 4 post-sleep onset (units=minutes)	32.86 (23.03)	26.85 (25.69)
REM post-sleep onset (units=minutes)	89.33 (28.24)	100.39 (26.42)
WASO (units=minutes)	26.70(32.5)	26.58 (21.86)
TST (units=minutes)	407.48(46.4)	381.02 (45.55)
REM Latency (units=minutes)	84.45(40.96)	63.95 (37.30)
PSOSE (0 to 1)	0.94 (0.08)	0.93 (0.06)
Alpha^a^	−1.86 (2.52)	−2.85 (3.66)
Beta^a^	−3.98 (2.62)	−5.28 (3.53)
Delta^a^	−4.51 (4.34)	−5.61 (5.06)

Means (SD) for continuous variables and counts (%) for sex

*MADRS* Montgomery-Asberg Depression Rating Scale, *HAM-D* Hamilton Depression Rating Scale, *SSI5* Scale for Suicide Ideation-5, *HAM-A* Hamilton Anxiety Rating Scale, *WASO* wakefulness after sleep onset, *NREM* non-rapid eye movement sleep, *TST* total sleep time, *PSOSE* post-sleep onset sleep efficiency

^a^Normalized, on dB scale, and averaged over the 5.04 h post-onset time period

### Study design and clinical ratings

**Fig. 1:**
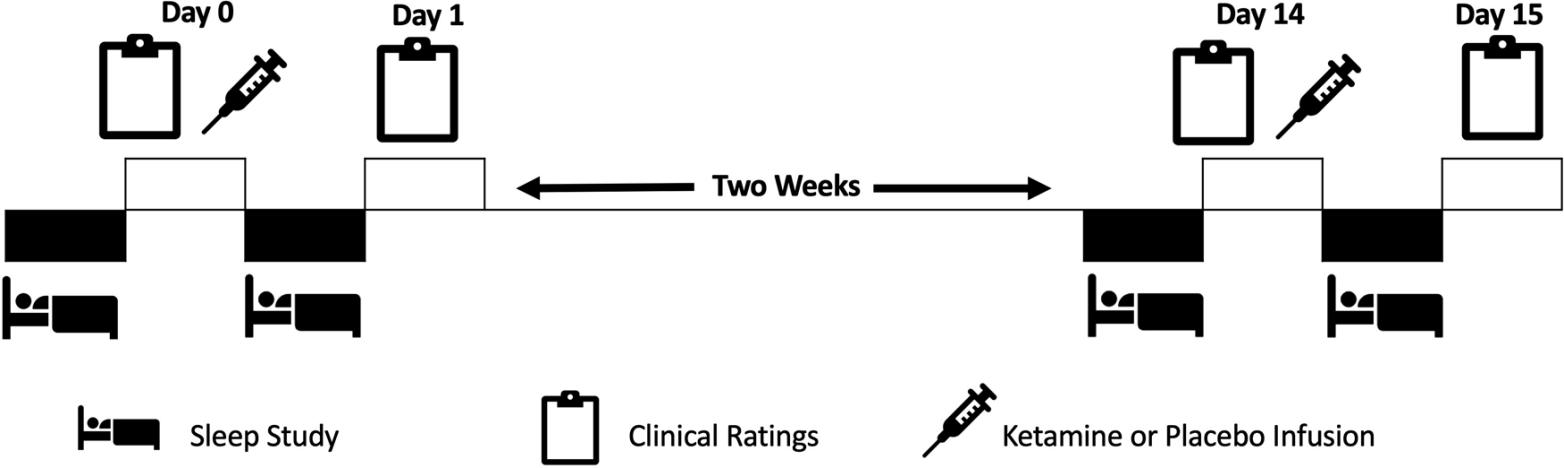
Study Design. Schematic of randomized crossover ketamine clinical trial compared to saline placebo with sleep studies.

Participants were randomized to receive either ketamine or saline placebo as a first infusion; after two weeks, they received the other dose in the second crossover period. After an initial adaptation PSG night, participants underwent PSGs the night before (baseline) and the night after (post-infusion) both ketamine and placebo infusions (see Fig. 1). Baseline clinical ratings were collected in the morning after completing the baseline PSG but at least one hour before ketamine or placebo infusion. Post-intervention ratings were collected approximately 24 h after each infusion (placebo and ketamine). Depressive symptomatology was measured via Montgomery-Asberg Depression Rating Scale (MADRS) total score with the suicide item removed (MADRS^*^) [28]. The Hamilton Depression Rating Scale (HAM-D) [29] was used as another measure of depressive symptoms as well as for single items representing early, middle, and late insomnia (scaled from 0 to 2, with 2 representing the most severe insomnia). Anxiety was measured via the Hamilton Anxiety Rating Scale (HAM-A) [30]. SI was quantified using a weighted average of one MADRS and two Beck Depression Inventory (BDI) [31] suicide items identified by a previously published exploratory factor analysis [32] as well as the first five items from the Scale for Suicidal Ideation (SSI-5) [33].

To test the hypotheses related to baseline diagnostic differences, all baseline PSGs (i.e., from both crossover periods) from both HV and TRD participants were used. To test our ketamine-related hypotheses, only the baseline and post-infusion PSGs *from the TRD sample* were included because these hypotheses only pertained to the TRD participants.

### PSG and power spectrum estimation

Overnight PSG was conducted in a sleep laboratory at the NIH using a Nihon-Kohden system (Neurofax version 05-50, Japan) and Polysmith Acquisition and Review Software (version 4.0.25.0). Electrooculograms and submental electromyograms were used to evaluate eye movements and muscle activity, respectively. All sleep recordings in the current study underwent a quality control process that entailed reviewing the record for technical problems. Of the sleep recordings initially considered for our study, five were removed for various technical reasons (e.g., incorrect voltage, person pulled off leads, pervasive sweat-related artifacts), resulting in the final sample used in the analysis^1^. All participants were instructed to maintain their usual sleep schedule and to refrain from napping while participating in the study.

Following each PSG session, trained raters (N.H., W.D.) who were blind to diagnosis and treatment assigned a sleep stage (rapid eye movement (REM), wake, movement, or non-rapid eye movement (NREM)) to each 30-second epoch between Lights-Out and Lights-On. Participants selected a Lights-Out time near 2300 h ± 1.0 h, consistent with their home sleep schedule; the latest Lights-On time was 0700 h. All sleep stage assignments were done using guidelines established by Rechtschaffen and Kales [34]. These stages were then used to calculate the following sleep macroarchitecture measures of arousal: wakefulness after sleep onset (WASO; hours awake after sleep onset and before Lights-On), total sleep time (TST; hours of sleep after sleep onset and before Lights-On), REM latency (minutes from sleep onset to first epoch of REM), and Post-Sleep Onset Sleep Efficiency (PSOSE) (TST/time between sleep onset and Lights-On). Other common sleep macroarchitecture measures were also calculated to provide descriptive information about the sample (see Table 1).

Functional data included normalized alpha, beta, and delta power measured at 605 30-s epochs (5.04 h) starting at sleep onset (first NREM stage 2 epoch). Alpha and beta bands were selected due to their previously discussed associations with wakefulness, and delta power was included because it is the inverse of wakefulness. The Supplement contains further details on the calculation and normalization of power spectrum estimation.

### Statistical methods: Alpha, beta, and delta power temporal dynamics

#### Baseline diagnostic differences

A functional data analysis approach was adopted to investigate electroencephalograhy (EEG) power effects as smooth functions of time [35]. Specifically, multilevel function-on-scalar regression (FoSR) [35–37], was used to examine baseline diagnostic differences in the function of normalized alpha, beta, and delta power over the five-hour post-sleep onset interval. FoSR has the general form:

1 $${y}_{{ij}}(t)={X}_{{ij}}^{T}\beta (t)+{b}_{i}({\rm{t}})+{\varepsilon }_{{ij}}\left(t\right)$$

where *i* is the individual, *j* is the infusion number (1 or 2), ${y}_{{ij}}(t)$ represents power as a function of time (observed finitely at many time points t = 1 to T), ${X}_{{ij}}^{T}$ is a row vector containing values of *q* predictors for person *i* at infusion *j*, $\beta (t)$ contains the *q* corresponding coefficient functions, ${b}_{i}(t)$ is a time-varying random subject effect, and ${\varepsilon }_{{ij}}\left(t\right)$ is a randomly distributed functional error term. This approach is similar to the usual regression model except that the outcome is a function over time that preserves the dynamic nature of the sleep process and allowed us to determine if and how a variable of interest was related to the temporal dynamics of the outcome. For example, in traditional regression, diagnostic differences are estimated in beta power at one hour from sleep onset in one model and diagnostic differences in beta power at 2 h from sleep onset using a separate model. In contrast, FoSR employs a single model that accounts for the residual correlation over time and provides smooth estimates of the diagnostic differences along the time domain. To interpret diagnostic differences in the model, the Fast Univariate Inference (FUI) approach was used, which is scalable to large-scale, high-dimensional data by first fitting massively univariate mixed models then smoothing estimates along the time domain [37]. Confidence bands (95%) are used for joint statistical inference and account for correlation among time points and within persons, rather than p-values. The FUI method is implemented in the R package *fastFMM* [37], available on CRAN.

#### Ketamine’s effects in TRD

The approach described above was also used to assess whether ketamine affected oscillatory patterns in arousal-related frequency bands differently than placebo.

#### Mediation analyses in TRD

The Barron-Kenny (BK) approach [38] was used to separately test the hypotheses that ketamine-related effects on alpha, beta, and delta power over time would mediate its therapeutic effect on SI. The BK mediation approach required testing: (1) whether ketamine had a therapeutic effect on SI; (2) whether ketamine was associated with the EEG spectral power function; and (3) whether the spectral power function was associated with SI. The first hypothesis was tested using Day 1 post-infusion SI score as the outcome in a linear mixed model (see Eq. (2) below) that included drug as the fixed effect of interest. The second hypothesis was tested using the above tests of ketamine’s effect on spectral power functions. The third hypothesis was tested using a scalar-on-function mixed effects regression model that included the main effects of power spectrum (e.g., alpha) and drug. As per the BK approach, if the effect of the power spectrum was significant and the effect of drug was significant, the null hypothesis of no partial mediation could be rejected. If the effect of the power spectrum was statistically significant and the effect of drug was not statistically significant, results indicate that complete mediation could not be rejected; additional details on this model are available in the Supplement.

### Statistical methods: macroarchitecture

#### Baseline diagnostic differences

Baseline diagnostic (MDD vs HV) differences in WASO and TST were tested using a linear mixed model that took the general form:

2 $${Y}_{{ij}}={X}_{{ij}}^{T}\beta +{b}_{i}+{\varepsilon }_{{ij}}$$

where *i* represents the participant, *j* represents infusion number (or period) (1 or 2), ${b}_{i}$ is a random intercept, and ${\epsilon }_{{ij}}$ is a normally distributed error term. ${X}_{{ij}}^{T}$ represents the row vector of predictor variables; for the baseline models, these included infusion number, age, diagnosis, and sex (the latter three did not depend on *j*). $\beta$ is a column vector that contains the estimated coefficient corresponding to each variable in ${X}_{{ij}}$. WASO was right-skewed and log transformed, and because TST was left-skewed, it was subtracted from eight (so that all values remained positive) and then log transformed.

Baseline diagnostic differences in PSOSE were assessed using a beta-distributed generalized linear mixed model with a logit link (beta GLMM) [39, 40] and a random intercept per person. The model was chosen because the beta distribution is a priori the most appropriate distribution for responses that are essentially continuous but strictly bounded between 0 and 1. For REM latency, diagnostic differences in time to first post-sleep onset REM epoch were modeled using a Cox proportional hazards model with robust standard errors to account for the two nights per person. Age, sex, and infusion number were also included as covariates in the PSOSE and REM latency models.

#### Ketamine’s effects in TRD

The effects of ketamine (relative to placebo) on WASO (log transformed as above) and TST in TRD were tested using linear mixed models (as described above). For PSOSE, a beta-distributed generalized linear mixed model (as described above) was used to test the effect of drug, and for REM latency, drug differences in time to first post-onset REM epoch were tested using a Cox proportional hazards model with robust standard errors (as above). For all models, covariates included age, sex, infusion number, period-specific baseline values, and within-person average baseline (to avoid cross-level bias) [41].

#### Mediation

Linear mixed models (see Eq. (2)) that each included a drug x post-infusion sleep macroarchitecture measure were used to test the hypothesis that treatment-related effects on macroarchitecture measures mediated ketamine’s therapeutic effects on SI at post-infusion Day 1. Models also included age, infusion number, sex, period-specific baseline SI, and average baseline SI as covariates, as well as a random intercept per person. An identical approach was used for MADRS* total score.

Due to the exploratory nature of this study, no correction for multiple comparisons was made, and the risk of false negatives was minimized at the expense of incurring a higher false positive risk. All statistical analyses were performed in R [42].

## Results

### Baseline diagnostic differences

#### Alpha, beta, and delta power temporal dynamics

**Fig. 2:**
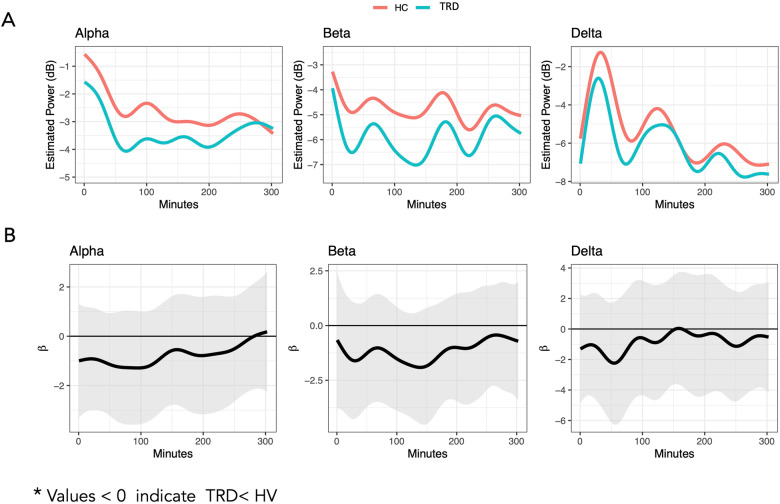
Comparison of Individuals with Treatment Resistant Depression (TRD) as Compared to Healthy Volunteers (HV). Estimated smooth curves (**A**) and differences with 95% joint confidence bands (**B**) for individuals with TRD and HVs. Smoothed diagnostic differences over time (in black) with the 95% joint confidence bands (in gray).

Multilevel FoS model results are presented visually in Fig. 2. Smoothed diagnostic differences over time suggested a general pattern of lower alpha, beta, and delta power in the TRD group that was more consistent during the first part of the five-hour post-onset interval; however, because the 95% joint confidence bands consistently contained zero (Fig. 2B), the differences are not considered significant.

#### Macroarchitecture

Baseline TST was lower in the TRD group than in the HV group (*b* = 0.36(SE = 0.12), *p* = 0.006, approximated Cohen’s f^2^ = 0.14) [43]. An association was observed between diagnosis and REM latency, with a greater event hazard in TRD (*b* = 0.48(SE = 0.20), *p* = 0.04) and shorter time to first REM epoch (REM latency) in TRD (median time to first REM epoch: TRD = 55 min; HVs = min). Diagnostic differences were not evident in either WASO (*b* = 0.005(SE = 0.19), *p* = 0.98) or PSOSE (*b* = 0.01(SE = 0.18), *p* = 0.94) (see Table 1 for raw means and standard deviations).

### Ketamine’s effect on sleep metrics in TRD

#### Alpha, beta, and delta power temporal dynamics

**Fig. 3:**
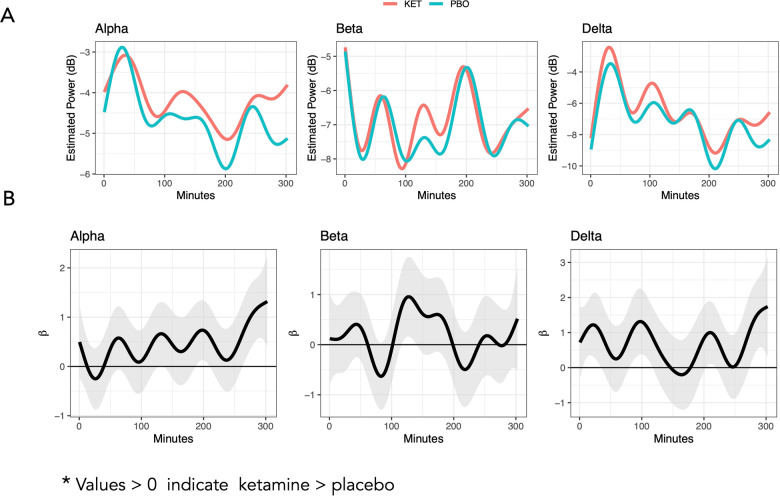
Comparison of Post-Ketamine versus Post-Placebo. Estimated ketamine and placebo smooth curves (**A**) and differences with 95% joint confidence bands (**B**). Smoothed drug effects over time (in black) with the 95% joint confidence bands (in gray).

Multilevel FoS model results are presented visually in Fig. 3. Smoothed drug differences over time, along with 95% joint confidence bands, supported significant ketamine-related increases on EEG power in all three frequency bands (Fig. 3B). For delta power, ketamine was associated with a recurring pattern of significant power increases throughout the post-sleep onset period. Recurring increases were also evident in alpha, with significant increases detected at the end of the five-hour period. In contrast, ketamine’s effect on beta power was associated with significant power increases in the middle of the time period.

#### Macroarchitecture

Ketamine had no effect on any of the macroarchitecture measures WASO: *b* = 0.21(SE = 0.14), *p* = 0.16; TST *b* = −0.14(SE = 0.11), *p* = 0.23; PSOSE (*b* = −0.12(SE = 0.14), *p* = 0.41) or REM latency (*b* = 0.11(SE = 0.28), *p* = 0.61); raw means and standard deviations can be found in Supplemental Table S1.

### Mediation of ketamine’s effect in MDD

#### Alpha, beta, and delta power temporal dynamics

Consistent with results reported in the primary publication from which this subset of participants was drawn [6], an expected ketamine-related decline was observed in Day 1 SI (*p* = 0.01) and Day 1 MADRS* one day post-infusion (*p* < 0.01). However, the results did not support ketamine’s anti-SI and antidepressant effects as being mediated by any functional variables, although there was a trend (*p* = 0.06) for ketamine’s antidepressant effects on delta power (see Table 2 for estimated relationships between clinical outcomes and post-infusion functional variables).

**Table 2:** Mediation analyses of suicidal ideation and antidepressant response to ketamine by (A) sleep macroarchitecture variables and (B) alpha, beta, and delta power temporal dynamics.

A	Linear mixed model results: interaction coefficients (standard errors) and *p* values		
Outcome	Mediation effect	Mixed model interaction coefficient (standard error)	*p* value
Suicidal Ideation	WASO x treatment	0.04 (0.09)	0.68
	TST x treatment	0.01 (0.04)	0.77
	PSOSE x treatment	−0.29 (0.62)	0.65
	REM latency x treatment	0.0002 (0.0004)	0.65
MADRS^a^	WASO x treatment	3.57 (5.85)	0.54
	TST x treatment	3.05 (3.09)	0.33
	PSOSE x treatment	−28.64 (39.31)	0.47
	REM latency x treatment	0.006 (0.03)	0.84
**B**	Scalar-on-function mixed effect regression results: tests of significance for smooth post-infusion EEG spectral power functions’ relationship with clinical outcome		
Outcome	Time-varying effects of EEG spectral power functions on clinical outcome		Scalar-on-function-regression results
	Frequency band		*p*
Suicidal Ideation	alpha	Post-ketamine	0.50
		Post-placebo	0.40
	beta	Post-ketamine	0.54
		Post-placebo	0.90
	delta	Post-ketamine	0.09
		Post-placebo	0.75
MADRS^a^	alpha	Post-ketamine	0.44
		Post-placebo	0.56
	beta	Post-ketamine	0.91
		Post-placebo	0.98
	delta	Post-ketamine	0.06
		Post-placebo	0.34

For details on statistical models, see statistics section and Supplementary Materials.

*WASO* wakefulness after sleep onset, *NREM* non-rapid eye movement sleep, *MADRS*^a^ Montgomery-Asberg Depression Rating Scale score with suicide item removed, *TST* total sleep time, *PSOSE* post-sleep onset sleep efficiency.

#### Macroarchitecture

The drug x post-infusion macroarchitecture sleep measure interaction was not significant for TST, WASO, PSOSE, or REM latency (*p* > 0.05 for all mediation effects; Table 2).

## Discussion

This study evaluated sleep-related arousal at baseline and following ketamine administration in unmedicated individuals with TRD and HVs enrolled in a randomized, placebo-controlled, crossover trial. At baseline, the hypothesized shorter REM latency and TST were observed in unmedicated individuals with TRD relative to HVs. Contrary to our hypotheses, however, unexpectedly *lower* baseline alpha, delta, and beta power were observed in the TRD group relative to HV group, though these findings were not statistically significant. Ketamine’s impact on sleep-related brain electrophysiology included temporally specific increases in delta, alpha, and beta power; the latter two effects were contrary to our hypotheses. Specifically, post-ketamine (vs placebo) delta power was significantly greater at regularly occurring intervals during the five hours under study, while post-ketamine alpha power was higher at the end of the night, and post-ketamine beta power was higher in the middle of the night. Ketamine exerted no detectable effects on any of the macroarchitecture measures such as PSOSE or WASO. None of the sleep variables mediated ketamine’s impact on depression or SI. Overall, our results underscore the impact of ketamine on the sleeping brain in TRD, as well as the use of functional data analysis (FDA) for understanding sleep electrophysiology following rapid-acting antidepressant treatment.

Collectively, the findings support the use of FDA to represent the sleep process as a temporally dynamic spectral power signal. The sensitivity of this approach may be maximized because information is not collapsed over time or within the confines of a defined state, thereby gaining proximity to the symphonic neural sleep processes managing the sleep-arousal balance. All EEG epochs are included in the analysis, including brief movements and more extended wakefulness periods that might have been excluded from more traditional sleep staging approaches. Also, in support of the utility and validity of FDA as applied to sleep EEG, patterns in the predicted values from the functional models were in line with what is known about sleep electrophysiology. Specifically, alpha and delta power demonstrated similar temporal patterns (see Figs. 2A and 3A) that tended to be inverted relative to temporal patterns in beta power [44]. Additionally, delta power findings in the present study were in line with previous findings of ketamine increasing slow wave activity in the first NREM episode [8]. Other areas of brain research have also supported the use of FDA, including studies using anatomic magnetic resonance imaging (MRI) and functional MRI measures [45, 46]. Because our application of FDA in a study of drug effects on sleep EEG is novel, additional work is needed to replicate these findings in other datasets and treatments to determine whether the potential effects are ketamine-specific or represent a signature of antidepressant response to treatment.

Our findings are also particularly relevant for understanding ketamine’s neurobiological mechanisms relative to sleep deprivation therapy as a non-pharmacologic rapid-acting antidepressant treatment [9, 47]. Specifically, the ketamine-related increases in alpha and delta power observed here are consistent with reports of alpha and delta power increases in recovery sleep following sleep deprivation in samples of HVs and individuals with seasonal affective disorder [48–50]. Such increases in alpha and delta power may reflect cortical excitability and brain plasticity processes that are hypothesized to underlie rapid-acting antidepressant mechanisms [9]. Ketamine has also been linked to changes in TRD wrist actigraphy markers of circadian rhythms [51]. Because individuals with MDD have been shown to have altered clock gene expression [52], it is relevant to note that both ketamine and 36-hour sleep deprivation therapy have been linked to changes in clock gene expression, including *Ciart, Per2, Npas4, Dbo*, and *Rorb* [53]. This further supports the possibility of shared mechanisms of action between ketamine and sleep deprivation therapy, including the role of clock gene expression. Because daytime, rather than nocturnal, biomarkers are often the focus of ketamine research [54], the current findings reinforce the need to take a systemic, 24-hour approach to studying ketamine’s antidepressant effects.

The baseline diagnostic spectral power function comparisons warrant further discussion, as the results for each of the three frequency bands were inconsistent with our hypotheses. First, the lower but nonsignificant group difference in delta power functions over time was qualitatively consistent with our hypothesis (lower delta in individuals with TRD vs HVs), and previously published literature supporting lower delta power—reflecting lower sleep drive—in depression. It is not clear if our failure to reject the null was due to sample effects, factors related to inpatient psychiatric hospitalization, or other methodological issues. Baseline diagnostic differences in beta and alpha power were also non-significant and qualitatively reversed relative to what the original hypotheses. While this could be due to methodological issues such as those mentioned above, an alternative explanation lies in reports supporting a *general lowering of power* in depression, even in frequency bands associated with wakefulness [55–57]. Further clarification of the nature and scope of the EEG signature of sleep and sleep drive in depression is recommended.

The absence of mediation effects highlights the challenges of identifying neurobiological moderators or mediators of clinical response to rapid-acting interventions. While other trials of repeated ketamine administration in individuals with TRD found that improvements in depression and SI were partially mediated by improvements in self-reported insomnia symptoms, these trials relied on subjective indicators of sleep quality rather than PSG-based measures [58, 59]. It is possible that sleep changes may be implicated in the durability of response to ketamine; however, this analysis was not designed to answer this consideration, and informal correlations between sleep macroarchitecture variables and MADRS scores seven days post-ketamine administration did not support a relationship. In general, the field has struggled to link neurobiological markers with clinical antidepressant response to ketamine. For instance, meta-analyses across ketamine trials found few consistent clinical moderators or neurobiological mediators of antidepressant response to ketamine [60, 61]. Much larger sample sizes may be required to power these types of moderation or mediation analyses, especially given that some trends in our analysis did not achieve statistical significance (e.g., the relationship between antidepressant effect and delta power) as well as the fact that changes in sleep-related arousal are likely only one correlate of the larger, systemic response to ketamine.

Limitations of the study include using data from a clinical trial powered to detect large effect sizes, which restricted our ability to detect small or medium effects of a specific biomarker. An additional limitation is that the TRD participants were primarily inpatients over the course of the study while the HV participants were members of the local community who spent most of their time on pass/off the unit and thus had more flexibility in terms of choosing a bedtime and adhering to their usual sleep patterns as requested. This difference in daily schedules is inextricably confounded with group and may have affected the results in a manner that cannot be disentangled from diagnosis. Fortunately, actigraphy data were also collected for 19 HVs, and actimeter (Actiwatch AW64; Philips, Amsterdam, the Netherlands) data could be screened to examine potentially unusual sleep behavior (e.g., staying up all night) in the three to four night(s) before baseline PSGs. However, when the six individuals who appeared to have alterations to their usual sleep patterns prior to PSG were removed, the same pattern of diagnostic differences in the macroarchitecture variables was observed. This suggests that more time off the unit was not necessarily a primary factor driving our diagnostic results. Additionally, there were no self-reported sleep measures beyond the insomnia items from the depression rating scales; future analyses should use scales such as the Pittsburgh Sleep Quality Index [62] to identify the association between oscillations in alpha, delta, and beta power with self-reported sleep quality. Lastly, this sample comprised individuals with severe TRD; future analyses should consider including a range of depression severity and/or other psychiatric diagnoses implicated in both sleep and suicide risk, including bipolar disorder and post-traumatic stress disorder.

Although not a limitation, per se, it should also be noted that the FDA approach used in the present study allowed the activity of alpha, beta, and delta frequency bands to be evaluated as functions over time, independent of REM/NREM sleep stages. Therefore, results should not be considered an effort to replicate our previous slow wave activity (SWA) findings [8] because those depended on boundaries of REM/NREM episodes to calculate SWA power.

A primary strength of this study is that its data were drawn from a placebo-controlled, double-blind, crossover ketamine trial and allowed each TRD participant to serve as their own treatment control. In addition, because all TRD participants underwent an inpatient medication washout prior to their first PSG, there were no confounding effects of antidepressant medication. Finally, HVs with PSGs provided important context for the inherent differences in sleep quality in individuals with TRD.

In summary, this study used an innovative FDA approach to PSG data and found that ketamine was associated with temporally-dependent changes in alpha, delta, and beta power. While these sleep changes did not mediate ketamine’s antidepressant effects, the results reinforce the need to consider sleep as a critical variable in understanding the impact of ketamine across neurobiological dynamics and systems.

### Supplementary information

Supplement

## Data Availability

The data that support the findings of this study are available from the corresponding author upon request.

## Footnotes

1 Three individuals (one HV, two with TRD) who did not have a PSG adaptation night were included in the sample. When sleep microarchitecture indices (TST, WASO, PSOSE, REM latency) were examined for individuals without an adaptation night, no pattern emerged implicating generally worse sleep during their first baseline PSG versus their second baseline PSG or in comparison to other individuals.
