# Supplementary material for: Functional changes in sleep-related arousal after ketamine administration in individuals with treatment-resistant depression
Source: Transl Psychiatry. 2024 Jun 4;14:238. doi: 10.1038/s41398-024-02956-2 (PMC11150508; doi:10.1038/s41398-024-02956-2)
Supplement: Supplementary file 1 — Supplement [file 41398_2024_2956_MOESM1_ESM.docx]

*Ballard et al — Functional Changes in Sleep-Related Arousal after Ketamine Administration*

*in Individuals with Treatment-Resistant Depression*

**Supplemental Methods**

*Spectral Estimation*

To estimate alpha, beta, and delta power, electroencephalography (EEG) recordings were selected from the two central leads (C3-A2, C4-A1) from a six-channel EEG montage. PSG values were calculated using the multitaper method available in Luna (<http://zzz.bwh.harvard.edu/luna/>), which has been shown to be less noisy and more accurate than periodogram-type methods that average over time and/or use single taper window functions. Fifteen tapers and a time half-bandwidth product parameter of 29 were used because the focus was on overall patterns rather than finer-grained fluctuations at higher temporal resolutions (2). For each frequency band (alpha (>8.0 to < 12.0 Hz), beta (>18.0 to < 26.0 Hz), and delta (>0.6 to < 5.0)), the power was averaged over the corresponding frequencies at each epoch starting at sleep onset (defined as the first epoch of stage 2 sleep; sample average onset at baseline=39.9 minutes (SD=35.3)) and spanning 605 30-second epochs (5.04 hours) of post-sleep onset recordings. This cutoff was chosen to ensure high coverage in the early morning hours while maximizing the number of PSGs included in our study. Thus, each time series of alpha, beta, and delta power for each person at each baseline/pre-infusion or post-infusion night was aligned at sleep onset and was 605 epochs, or 5.04 hours, long.

Lastly, for each time series, the alpha, beta, and delta power spectra were normalized. First, for a given time series, the average power was calculated per band (alpha, beta, and delta) over the 5.04 hour post-sleep onset time period. Then, for each epoch, alpha, beta, and delta power were divided by the respective (5.04 hour) average band power. This step was done separately for the right (C4-A1) and left (C3-A2) central leads and then averaged due to their high correlation. As a final step, to reduce skewness, the normalized power was transformed to the decibel scale ($10 x log10(normalized power)$) separately for each epoch and band. For a discussion of the need for normalization, see (3).

*Mediation model:* A scalar-on-function mixed effects regression model was used to test the hypothesis that spectral power mediates ketamine’s therapeutic effect (suicidal ideation (SI) or Montgomery-Asberg Depression Rating Scale (MADRS)). In this model, spectral power over time (alpha, for example) was used as a functional predictor, with the constraint that the effect varied smoothly across time points. The formula is shown below:

$y_{ij}= X_{ij}^{T}\gamma+\int\beta\left( t \right)W_{ij}\left( t \right)dt+d_{i}+\epsilon_{ij},$ (1)

where *i* is the individual and *j* is the infusion number (0 for the first infusion and 1 for the second infusion). For each individual at each infusion, $y_{ij}$ represents the outcome (Day 1 post-infusion SI factor scores) and $W_{ij}\left( t \right)$ represents the functional predictors along the functional domain (alpha, beta, and delta power over time). Models were fit separately for each band, and $\beta\left( t \right)$ represents the functional fixed effects of the spectral power in a particular band on outcome over time. The covariates $X_{ij}$ represent non-functional predictors, including infusion, drug, age, sex, period-specific baseline SI, and average baseline SI for the mediation model. A random intercept $d_{i}$ was included to account for within-person dependence, and the error term was denoted as $\epsilon_{ij}$ and assumed to be independent N(0,\sigma^2_\epsilon) errors.

**Supplemental References**

1. Rechtschaffen A. KA. A Manual of Standardized Terminology, Techniques and Scoring System for Sleep Stages of Human Subjects. Washington, D.C: Public Health Service, US Government Printing Office; 1968.

2. Prerau MJ, Brown RE, Bianchi MT, Ellenbogen JM, Purdon PL. Sleep neurophysiological dynamics through the lens of multitaper spectral analysis. Physiology (Bethesda). 2017;32(1):60-92.

3. Crainiceanu CM, Caffo BS, Di CZ, Punjabi NM. Nonparametric signal extraction and measurement error in the analysis of electroencephalographic activity during sleep. J Am Stat Assoc. 2009;104(486):541-55.

Supplemental Table S1: Differences in sleep architecture after ketamine compared to saline placebo

|  | WASO | TST | REM Latency | PSOSE |
| --- | --- | --- | --- | --- |
| Ketamine | 25.77 (29.66) | 402.96 (44.13) | 61.96 (29.90) | 0.94 (0.07) |
| Placebo | 36.85 (98.99) | 400.24 (44.64) | 55.49 (25.19) | 0.93 (0.10) |

Units are minutes except for PSOSE, which is a percentage.

WASO: wakefulness after sleep onset; TST: total sleep time; REM: rapid eye movement; PSOSE: post-sleep onset sleep efficiency
